# Social network support and harm reduction activities in a peer researcher-led pilot study, British Columbia, Canada

**DOI:** 10.1186/s12954-020-00401-3

**Published:** 2020-08-21

**Authors:** Sulaf Elkhalifa, Ehsan Jozaghi, Samona Marsh, Erica Thomson, Delilah Gregg, Jane Buxton, Ann Jolly

**Affiliations:** 1 School of Public Health, University of Ottawa, Room 101, 600 Peter Morand Crescent, Ottawa, Ontario KIH 8 M5 Canada; 2 School of Population and Public Health, University of British Columbia, 2206 East Mall, Vancouver, British Columbia V6T 1Z3 Canada; 3 Vancouver Area Network of Drug Users, 380 East Hastings Street, Vancouver, British Columbia V6A 1P4 Canada; 4 British Columbia/Yukon Association of Drug War Survivors, 380 East Hastings Street, Vancouver, British Columbia V6A 1P4 Canada; 5 Sex Workers United Against Violence, 334 Alexander Street, Vancouver, British Columbia V6A 1C3 Canada; 6 Western Aboriginal Harm Reduction Society, 380 East Hastings Street, Vancouver, British Columbia V6A 1P4 Canada; 7 University of Fraser Valley, 33844 King Road, Abbotsford, British Columbia V2S 7 M8 Canada; 8 The Fraser Health Authority, Suite 400, Central City Tower, 13450 – 102nd Avenue, Surrey, British Columbia V3T 0H1 Canada; 9 British Columbia Centre for Disease Control, 655 West 12th Avenue, Vancouver, British Columbia V5Z 4R4 Canada

**Keywords:** Social network support, Harm reduction, Chain link sampling, Drug use disorder, Canada, Community-based research

## Abstract

**Background:**

People who smoke drugs (PWSD) are at high risk of both infectious disease and overdose. Harm reduction activities organized by their peers in the community can reduce risk by providing education, safer smoking supplies, and facilitate access to other services. Peers also provide a network of people who provide social support to PWSD which may reinforce harm reducing behaviors. We evaluated the numbers of supportive network members and the relationships between received support and participants’ harm-reducing activities.

**Methods:**

Initial peer-researchers with past or current lived drug use experience were employed from communities in Abbotsford and Vancouver to interview ten friends from their social networks who use illegal drugs mainly through smoking. Contacts completed a questionnaire about people in their own harm reduction networks and their relationships with each other. We categorized social support into informational, emotional, and tangible aspects, and harm reduction into being trained in the use of, or carrying naloxone, assisting peers with overdoses, using brass screens to smoke, obtaining pipes from service organizations and being trained in CPR.

**Results:**

Fifteen initial peer researchers interviewed 149 participants who provided information on up to 10 people who were friends or contacts and the relationships between them. People who smoked drugs in public were 1.46 (95% CI, 1.13-1.78) more likely to assist others with possible overdoses if they received tangible support; women who received tangible support were 1.24 (95% CI; 1.02-1.45) more likely to carry and be trained in the use of naloxone. There was no relationship between number of supportive network members and harm reduction behaviors.

**Conclusions:**

In this pilot study, PWSD who received tangible support were more likely to assist peers in possible overdoses and be trained in the use of and/or carry naloxone, than those who did not receive tangible support. Future work on the social relationships of PWSD may prove valuable in the search for credible and effective interventions.

## Highlights

- In a peer researcher-developed study, people who smoke street drugs asked friends who also smoked drugs about relationships between 10 of their friends.
- Participants who received tangible items from network members were more likely to assist others during overdoses using naloxone.
- Women who smoked street drugs and received tangible support were more likely to carry and be trained in the use of naloxone than those who did not receive tangible support.
- The number of supportive network members was not associated with harm reduction behaviors.

## Introduction

Sustained and chronic use of illicit drugs in Canada is linked to various adverse health events that contribute a substantial economic burden to the healthcare system and result in death and disability [11, 34]. Approximately 3% of the Canadian population report using cocaine or crack, ecstasy, speed, methamphetamines, hallucinogens, or heroin in the past year [19].

In response, harm reduction initiatives aimed at reducing harmful drug behaviors rather than halting drug use itself, have been adopted in various locations across Canada [45]. The most common harm reduction programs are needle exchange programs (NEP), supervised consumption facilities (SCF), and opioid substitution therapies. Few harm reduction programs specifically tailored to people who smoke drugs (PWSD) have been established. The safer crack use kit (SCUK) distribution programs and supervised smoking facilities (SSF) have played crucial roles in mitigating infection rates among PWSD, as well as communicating crucial information on the health risks of drug use, and the preventative measures and treatments available [22, 32]. Spearheading many of these harm reduction initiatives in British Columbia is the Vancouver Area Network of Drug Users (VANDU), a peer-led organization comprising individuals with lived drug experiences. VANDU invites community members to design and implement activities to promote safe drug practices in the Downtown East Side Community (DTES) [23].

While these programs may be extremely beneficial to PWSD [29], benefits can only be attained through attendance. Recent initiatives have focused on peer networks as a means of delivering harm reduction practices [18, 29, 41, 44]. Specifically, harm reduction behaviors recommended by official programs are transferred from highly connected individuals among PWSD to those who may otherwise not be exposed or inclined to attend formal programs and services. Last, social networks, including PWSD networks, not only transmit information but also harm reduction social norms and support, comprising entities with their own culture [27], the sum of which is greater than its parts [25].

Social support has been subdivided into three different kinds. Informational support is defined as the provision of information, guidance, advice, and suggestions to individuals in the hopes of assisting them in solving a problem. Tangible support is defined as direct, physical interaction such as providing material goods, money, or useful services. Emotional support is defined as interactions intended to lift the self-esteem of the recipient, such as letting recipients know that they are valued, accepted, and loved [5]. Studies have demonstrated considerable variability in the provision and reception of social support between men and women [2, 33]. Women are more likely to seek social support from network members than their male counterparts [43], which may indicate that women are more open and receptive to the positive benefits associated with social support than men. Moreover, women have been reported to perceive events as more stressful than men, even after adjusting for the stressful event in question [7]. Therefore, variability in the biopsychosocial mechanisms that mediate the beneficial effects of social may be a function of differences in perceptions of, and response to, stressful events between women and men.

No studies examining the impact of support in the social network of people who smoke illicit drugs were found. However, in the case of injection drug users, Stein et al. found that although the risk of needle sharing was lower in the presence of friendship in social networks of injection drug users, risky drug behavior among friends or families increased the risk of needle sharing [40]. Among large networks, the likelihood of needle sharing rose in relation to support network size, while in small networks, needle sharing was reduced in the presence of social support [42].. The authors speculate that the act of sharing needles is a form of bonding counteracted only by the social support of network members when networks are small.

Social exchange theory posits that individuals retain relationships on the basis of a cost-reward analysis, such that relationships that provide rewards at lower costs are selected and retained. In contrast, relationships whose maintenance incurs negative costs to the individual are discarded. In this way, “rules” on reciprocity govern the evolution of relationships [6]. However, it is possible that the surrounding environment may force social ties that may not be voluntary (such as ties with a drug dealer or pimp) by limiting the choices of potential contact. Therefore, social ties may function as a source of stress and anxiety [13]. The above example shows that the presence of close contacts in one’s social network is not synonymous with receiving social support.

During a pilot study to determine the feasibility of combining a Community Based Participatory Action Research (CBPAR) with respondent-driven sampling (RDS), participant-researchers collected information on their social network members (Elkhalifa submitted). Our main goal was to use data from the pilot to explore whether the social support of friends and contacts (received social support) was associated with (a) reported activities that reduce the risk of drug-related harm in PWSD; (b) greater personal network size of participants and; (c) greater reported access to health care services. Our hypothesis was that social support of any kind would facilitate or enable harm reduction practices, compared with individuals who reported no social support. We hypothesized also that people who reported larger social network size overall may be more likely to have social support members due to higher numbers of people in their network and consequently, a higher chance of having a supportive network member. Last, we also hypothesized that those who reported social support were more likely to have presented for a medical appointment with a physician or nurse.

## Method

Researchers used a Community Based Participatory Action Research (CBPAR) approach that involves the participation of the community in the development, implementation, and dissemination of research. By incorporating the inputs, needs, and concerns of the local organizations and the people living in these vulnerable situations, researchers are able to foster an environment of cooperation, co-learning, and collective action [46]. In this particular study, the British Columbia/Yukon Association of Drug War Survivors and Vancouver Area Network of Drug Users Board VANDU, Vancouver, British Columbia, together with researchers from the University of British Columbia defined the research questions, decided on a community-based participatory action research (CBPAR) approach and selected the respondent-driven sampling method in which people from hidden communities recruit each other. Respondent-driven sampling (RDS) is a method of sampling intended to reach “hidden” populations who may be involved in taboo or illicit activities [1], and who are unlikely to be represented in a sampling frame from which a random sample may be selected. Originally designed by Heckathorn, the method involves selecting initial respondents from the hidden population [20], for example, people who smoke drugs, and who are recognized by service providers and PWSD as knowing a lot of people within the group. They are invited to participate in the study, give consent, and answer a questionnaire. Each questionnaire usually contains a number of questions about immediate close contacts (about 5 or 10), in order to gain perspective on the people with which the participant socializes. After questionnaire completion, they are given uniquely numbered coupons, usually three, to give to three people who also smoke drugs. They then contact the study staff whose contact details are on the card, they may consent, be interviewed, and they are given three coupons in turn. The sample builds in “waves” of referrals until the required sample size is reached, though usually the number of waves (optimally around 7), is more important than generating a large sample of people. After several waves, the proportion of characteristics stabilizes between generations and diversifies from the characteristics of the initial seeds [1]. This “equilibrium” together with adjustments for the total network size of the respondent, and non-probability of selection, forms the basis for inferring the population characteristics from the non-probability RDS sample [21]. In other words, we can measure the unequal chances of a participant being selected in the sample, so we can adjust for it.

Consultations about the study with VANDU began in 2015 after Dr. Jozaghi and VANDU had completed a previous study. It was one of the members of VANDU who proposed researching the social networks of people who smoke drugs, as very little was known about them, but many came to VANDU to pick up pipes. Dr. Jozaghi wrote the grant proposal with a similar harm reduction approach in mind as the previous study with people who inject drugs. He then obtained his post-doctoral fellowship, and after he informed the board at VANDU, they began planning the new study. They immediately requested that, unlike the previous study where Dr. Jozaghi was paid to interview and collect data from people referred to him by VANDU, they wished to be paid as part of the post-doctoral fellowship research funding. From May to August, Dr. Jozaghi and the board met to refine the draft proposal. In the summer of 2016, Dr. Jozaghi and the staff and board of VANDU met at a restaurant at Dr. Jozaghi’s expense to comment on, and revise the questionnaires. They recommended shortening the questionnaires, and excluding questions on sexual behavior but including ones on opioids and naloxone. On their advice, Dr. Jozaghi focussed the project on the two sites, Vancouver and rural Abbotsford, where fewer services were available. The VANDU board put Dr. Jozaghi in touch with the BC-Yukon Association of Drug War Survivors (BCYADWS) who introduced him to counterparts with lived drug experience in Abbotsford. They and four members of the Western Aboriginal Harm Reduction Society provided him with feedback on the study details, implementation, training of interviewers (peer researchers), questionnaires, and consent forms. For example, Ms. Thompson and Ms. Marsh (coauthors) recommended Dr. Jozaghi pay peer researchers by the questionnaire rather than an hourly wage. They also supervised questionnaire completion and completed data entry, and were paid as research assistants to the project by the University of British Columbia. On completion of the training, the peer researchers went into their respective communities to interview participants. When data collection was over, Dr. Jozaghi gave them handwritten cards and a small donation to thank them for their work. In addition, they received an official certificate of appreciation from the British Columbia Centre for Disease Control. The peer researchers and VANDU board have reviewed our drafts and will review the current submission again with interest. It was they who recommended submission to Harm Reduction Journal as they understood that they and others would have free access to the paper.

Additional methods for this study have been described in detail [23]. Briefly, after consultations with two community groups above, the research proposal was approved, along with the questionnaire, and oral consent. Advertisements for peer researchers disseminated by community advertisements in Abbotsford, and posters distributed by community members in Vancouver focussed on facilitating the hiring of community members usually excluded from research. Eight and seven PWSD with lived-drug experiences were recruited from Abbotsford and Vancouver, respectively. These initial peer researchers were asked to recruit 10 “contacts or friends” from their established networks; who use illegal drugs mainly through smoking, were 19 years of age or older, and to whom they felt comfortable administering the questionnaire [23]. Besides providing information on themselves, each of the 10 friends then provided detailed proxy information on ten of their “friends or contacts” and the relationships between them, for a total of about 1500 people. Recruitment ended at the first wave of contacts or friends, due to time and resource constraints, and because this was an initial pilot to demonstrate feasibility rather than to provide definitive, generalizable results. Each peer researcher completed 10 h of training in applied ethics. Ethics approval for this research, which included approval of the consent to participate, was obtained from the University of British Columbia (Certificate H16-01580) and from the University of Ottawa (H-05-18-741). While smoking drugs excluding marijuana is illegal in Canada, there has been a long history of research in British Columbia and elsewhere in Canada where medical confidentiality is strictly maintained, and formal services such as safe consumption sites are officially approved [16]. Additionally, RDS was designed to allow participants to remain anonymous, and protected from the criminal justice system [20].

Because friends and contacts of an initial participant may also be friends and contacts of others, and because they were encouraged to use aliases in lieu of legal names, egocentric network sizes were determined using a hierarchical cross-network matching algorithm. The number of friends a participant nominated was added to the additional friends of any one in their network who cross matched with other nominated friends. The social networks of peer researchers were joined together in the same manner. Preliminary unique identifying data combined a mix of variables including the names/aliases, demographic attributes, and other variables. Then, the location and gender of suspected matches were compared. Successful matches were considered a match if they had ages within 10-year range; drug(s) of choice; current drug use status; and within a 5-year range of previous drug use [3]. Subjects that matched in at least three of the above mentioned variables were judged to be the same individual. Physical and mental health status and drug route of administration were used to verify matches and to resolve outstanding discrepancies.

### Dependent variables

Six harm-reducing behaviors, which we thought would be affected by a person’s social network included the following: using brass screens to smoke crack, being trained in CPR, having assisted other PWSD who have overdosed, being trained in naloxone use, and carrying naloxone. Because being trained in naloxone use and carrying naloxone were found to be highly correlated, they were combined into a single variable.

### Independent variables

The primary independent variables evaluated were network size and the presence of three types of support: informational, tangible, and emotional, from at least one person in one’s network (Table 1).

**Table 1:** Categorization of support roles into emotional, tangible, and informational support

Role number	Support role
**Emotional support**	
Role 15	Talked to me and asked how I was doing
Role 17	Came with me to hospital
Role 16	Know me by first name
**Tangible support**	
Role 4	Provided pipes, alcohol swaps, filters …
Role 9	Provided food, coffee, juice, or water
Role 19	Lent me some money or dope when I was dope sick
Role 10	Performed CPR when I/or someone overdosed
Role 11	Administered naloxone when I/or someone overdosed
Role 12	Called ambulance for help when I/someone overdosed
Role 14	Broke up fight
Role 21	Lent me some money for food when I was hungry
**Informational**	
Role 1	Told me about detox
Role 2	Taught me how to fix my pipe or my dope
Role 3	Told me about Insite/VANDU/or other harm reduction places
Role 5	Referred me to a nurse or a doctor
Role 6	Referred me to a homeless shelter
Role 7	Referred me to a place where I could get food
Role 8	Referred me to a pharmacy where I could get methadone
Role 13	Provided harm reduction education
Role 18	Referred me to a welfare office
Role 20	Referred me to a good dealer
Role 22	Told me where to get naloxone
Role 23	Showed me how to use naloxone

### Model building

Confounders with a significance level below 0.05, or that yielded a 10% or greater change in the coefficient of interest were retained, while variables that were insignificant were removed in a manual stepwise process. Gender and age were identified as important factors in the health outcomes of social support [5, 9, 38], and were retained in the models, regardless of their significance. Collinearity was reduced by selecting the most significant of highly correlated variables (chi-square/fisher exact test *p* value < 0.05), and eliminating other correlated variables. Lastly, interactions between all the main predictors of interest and all other variables, including those found to be insignificant in the preliminary effects model, were tested.

To account for the correlation between participants selected by the same peer researcher, a fixed effect generalized linear model procedure was used for all outcomes; missing data was accounted for using multiple imputations [48].

## Results

**Fig. 1:**
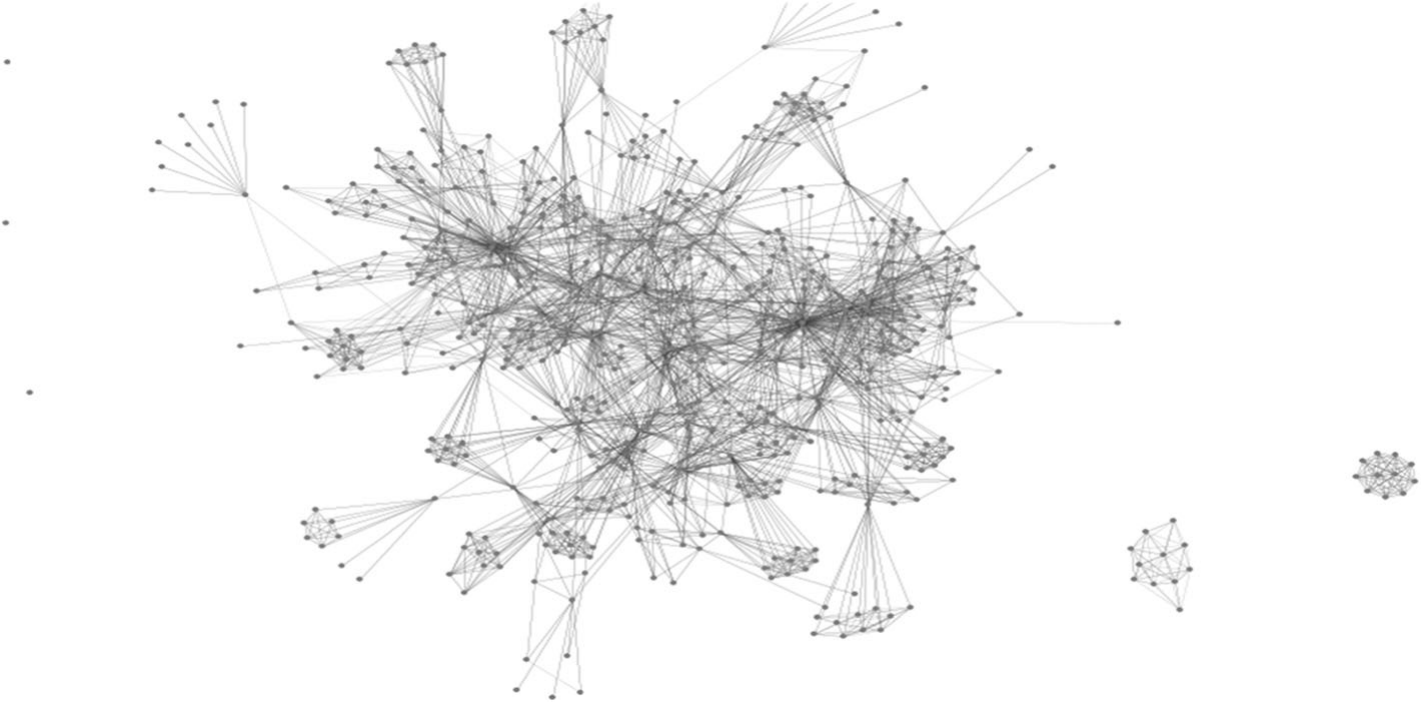
Social network of eight Abbotsford peer researchers, 79 recruits, and their 739 friends. Dots represent individuals, and lines between them represent relationships, including recruitment referrals into the study

**Fig. 2:**
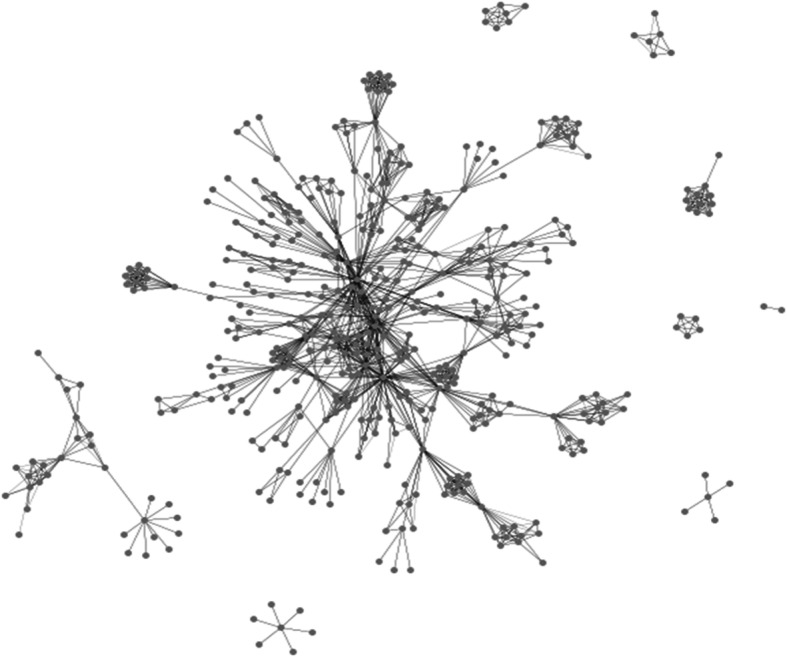
Social network of seven DTES peer researchers, 70 recruits, and their 498 friends. Dots represent individuals, and lines between them represent relationships, including recruitment referrals into the study

Eight of 10 and seven of eight peer researchers in Abbotsford and Vancouver successfully completed applied ethics training and recruited 79 and 70 friends or contacts (known as alters), who reported on 739 and 498 friends from whom they may receive support, respectively (Figs. 1 and 2). The demographic and behavioral attributes of the 149 friends and contacts of initial participant researchers are listed in Table 2. One contact or friend in Abbotsford did not complete their questionnaire and two identified as transgender, too small to be analyzed, so their genders were recorded missing and imputed.

**Table 2:** Distribution of participants’ characteristics in Abbotsford and Downtown Eastside (DTES), *n* = 149. Data presented as either count (percent frequency) or mean (standard deviation)

Demographic variables of participants	***n*** = 149, ***n*** or mean (% or SD)
Age	44 (11)
Male	71 (48)
Homeless	51 (34)
House/apartment	38 (26)
Living with friends or family	15 (10)
Supported living	45 (30)
First nations	62 (42)
Relationship status (single)	36 (24)
**Reported medical condition**	
HCV	59 (40)
No medical condition	61 (41)
Anxiety	79 (53)
Depression	84 (56)
No mental condition	25 (17)
Other mental conditions	72 (48)
**Drug use**	
Meth preferred	99 (66)
Crack preferred	71 (48)
Pipe source	
Outreach organizations	122 (82)
Store	30 (20)
Peers	21 (14)
Lend, borrow, or share pipes	84 (56)
Overdosed in the past month	15 (10)
Trained on how to use naloxone (naloxone)	100 (67)
Trained on CPR	102 (68)
Carry naloxone	70 (47)
Have assisted peers who have overdosed	66 (45)
Arrested for smoking or using illicit drugs in public	24 (16)
Received tickets for smoking or using illicit drugs in the past	12 (8)
Have experienced violence or exploitation when using drugs in public by:	
PWSD	83 (56)
Dealers	57 (39)
Police	48 (32)
Have experienced psychosis or paranoia as a result of smoking illicit drugs in the past	91 (61)
Have had blisters, cuts, damaged, or infections to your mouth, oral area, or lips in the last month	39 (26)
Number of days since visiting a doctor or a nurse	152 (436)
Public drug use	106 (71)
Pipe screen material	
Brillo	59 (40)
Brass	54 (36)
Meth equipment	48 (32)
**Network characteristics**	
Network size	21 (14)

^1^Pairwise deletion for missing values used

In bivariate analysis (Table 3), PWSD who had received informational support were 1.14 (*p* = 0.04) more likely to assist people who had overdosed. Those who received tangible support or informational support were 1.10 (*p* < 0.01), and 1.13 (p < 0.03) times more likely to be trained in the use of, and/or carry naloxone.

**Table 3:** Univariate generalized linear model of harm reducing behavior among people who smoke illicit drugs

Harm reducing behavior	Correlate	OR (± 95% CI)	SE	Pr > ChiSq
**Using brass to smoke crack**	Informational support	0.96 (0.87, 1.05)	0.04	0.33
	Tangible support	0.99 (0.93, 1.05)	0.03	0.65
	Emotional support	1.00 (0.91, 1.10)	0.05	0.96
	Network size	1.00 (0.98, 1.02)	0.01	0.87
**Acquiring pipes at outreach organizations**	Informational support	0.97 (0.84-1.10)	0.06	0.54
	Tangible support	1.07 (0.93-1.22)	0.07	0.33
	Emotional support	0.87 (0.72-1.04)	0.08	0.13
	Network size	1.00 (0.99, 1.02)	0.01	0.62
**Assisted overdosed peers**	Informational support	1.10 (0.99, 1.21)	0.06	0.06
	Tangible support	1.14 (1.04, 1.25)	0.05	0.04*
	Emotional support	1.11 (0.94, 1.28)	0.08	0.17
	Network size	1.00 (0.98, 1.02)	0.01	0.90
**Trained in CPR**	Informational support	1.01 (0.90, 1.13)	0.06	0.91
	Tangible support	1.07 (0.94, 1.21)	0.07	0.31
	Emotional support	1.02 (0.88, 1.17)	0.07	0.82
	Network size	1.03 (0.99, 1.06)	0.02	0.12
**Being trained in and/or carrying naloxone**	Informational support	1.13 (1.01, 1.27)	0.06	0.03*
	Tangible support	1.10 (1.02, 1.19)	0.04	0.01*
	Emotional support	1.00 (0.89, 1.12)	0.06	0.93
	Network size	1.00 (0.98, 1.02)	0.01	0.79

*Statistical difference *p* < 0.05

After including covariates which may be expected to affect the relationship between the independent variable and the dependent variable in the model, only the association between tangible support and assisting friends and being trained in and/or carrying naloxone persisted (Tables 4 and 5). Table 4 indicates that tangible support is associated with assisting others during an overdose, though with different relative probability, depending on whether the participants smoked drugs in public in the last month or not. There was an interaction between tangible support and public drug use, such that those who used drugs in public and received tangible support were more likely to assist others during an overdose than those who did not use drugs in public. An increase of one person providing tangible support in the networks of PWSD corresponded to an increase of 58% in the odds that they would assist their friends or contacts in an overdose. Likewise, those who received tangible support and were women, were 24% more likely to be trained in the use of and/or carry naloxone (Table 5). The model of correlates for assisting overdosed friends or contacts was adjusted for gender, age, public drug use, and number of years residing in Downtown Eastside. Being trained in, or carrying naloxone was adjusted for gender, age, trained in CPR, and number of days since visiting a doctor/nurse.

**Table 4:** Adjusted generalized linear model of assisting peers stratified by public drug smoking

Harm reduction	Correlate	Public drug smoking in the past month	OR (± 95% CI)	SE	Pr > ChiSq
Assist peers during overdose^a^	Tangible support	Yes	1.08 (0.99-1.17)	0.05	0.08
		No	1.46 (1.13-1.78)	0.17	< 0.01*

^a^Adjusted for age, gender, and number of years residing in Downtown Eastside

*Statistical difference *p* < 0.05

**Table 5:** Adjusted generalized linear model of being trained in and/or carrying naloxone stratified by gender

Harm reduction	Correlate	Gender	OR (± 95% CI)	SE	Pr > ChiSq
Trained in and/or carry Narcan^a^	Tangible support	Male	0.87 (0.73-1.00)	0.07	0.07
		Female	1.24 (1.02-1.45)	0.11	0.01*

^a^Adjusted for age, gender, CPR training, and number of days since last visiting a doctor or nurse

*Statistical difference *p* < 0.05

For an increase of one person providing tangible support in the network of female PWSD, there was an increase of 25% in the odds of being trained in and/or carrying naloxone.

We examined whether network size was associated with the number of contacts who provided *any* of the social support roles (Table 6). Bivariate analysis revealed a weak correlation between social support and network size that disappeared following adjustment for gender: age, lending, borrowing or sharing drugs, carrying naloxone, and the number of people known.

**Table 6:** Adjusted and unadjusted generalized linear model of the association between social support and network size

Harm reducing behavior	Correlate	OR (± 95% CI)	SE	Pr > ChiSq
**Unadjusted network size**	Social support	0.09 (1.01, 1.88)	0.05	0.04
**Adjusted network size**	Social support^a^	1.08 (0.99-1.17)	0.04	0.07

^a^Adjusted for age, gender, lending borrowing or sharing drugs, carrying naloxone, and number of people known

## Discussion

To our knowledge, this is one of the first studies examining the effect of social network support on naloxone carriage, training, or use among people who smoke drugs. We set out to examine the relationship between social networks and the harm reducing behavior of PWSD, and found that the probability of assisting a person during an overdose was associated with having a person in one’s network who provided tangible support, such as drugs, food, money, or administration of naloxone when the participant needed it. The odds of assisting contacts or friends during an overdose increased with each addition of a contact who provided tangible support in drug smokers’ network, but only for those who reported smoking in public areas in the past month. This is probably because PWSD in public are more likely to be exposed to overdosed friends or contacts, and therefore, more familiar with the procedure required to resuscitate them. Kerr et al. identified public drug use as a positive correlate of non-fatal overdose among poly-substance people who inject drugs (PWID) [24]. Another contributing factor is the fact that people who smoke in public rather than in private may be satisfying intense cravings for the drug elicited by withdrawal, and to avoid law enforcement and other individuals, PWID will rush their drug use when in public, consuming large amounts of drugs in a short period of time. This urgency is more likely to lead to mistakes in dose: omitting safer practices, less access to sterile supplies, and risk from public areas being unhygienic [4, 10]. In Germany, a survey showed that a large percentage of PWID will regularly consume drugs in locations near the area of purchase to end withdrawal symptoms [49].

Therefore, while PWSD in public areas are at a greater risk of overdose and less likely to smoke safely, they are more likely to be assisted by friends or contacts. A strong association between public drug use and homelessness was identified in our study (*p* < 0.01 data not shown), and in other studies [24], as homeless people do not have a private space in which to use drugs. Homelessness is also a risk factor for overdose and premature death, because of the multiple social and physical barriers encountered in everyday life [14, 15, 30].

The effect of tangible support on the likelihood of being trained in and/or carrying naloxone varied according to gender. Women who smoke drugs and who had tangible network support were more likely to carry, and be trained in the use of naloxone, whereas it did not seem to affect men. In a population similar to ours of women who inject street drugs, those who reported three or more sources of social support were less likely to have a non-fatal overdose than those who reported fewer sources of support [31].

The size of participant’s social network was not associated with the quantity of social support received among people who smoke illicit drugs, after adjusting for confounders. Similarly, previous studies have reported a weak correlation between the number of social ties and the availability of social support received [37, 39]. The lack of correlation may also be due to the fact that peer researchers reported on the numbers of contacts and their perceived social ties with friends and contacts, which may not reflect actual social ties. Relationships among our participants do not appear to be maintained purely for their anticipated future benefit, posited by social exchange theory. Participants reported assisting other PWSD with naloxone, not only their close friends or peers. Also, the predictor for doing this was training, and availability of naloxone having close social support members in their network who may return the favor was not associated with assisting others during an overdose.

We have shown how the presence of social support may affect both harm reduction and risk interactions. The risk environment including social networks in which PWSD find themselves is a crucial, yet often overlooked factor for understanding and predicting the harm production and reduction of drug use. In the context of harm reduction, the enabling environment comprises a wide array of micro and macro-level factors, all of which distill down to the interpersonal connections of individuals and their interactions with the environment ([8] [28];). As a consequence of this framework, drug-related risks can be perceived as a complex construct that is reflective of not only individual-level factors, but the interplay of those factors with the social environment [35].

The practical implications of these findings for harm reduction practitioners, policymakers, and others interested in addictions, are the provision of training, and naloxone itself will facilitate PWSD to provide assistance to each other to reduce overdoses. This is desirable for the following reasons: (1) they are more likely to be able to respond more promptly than any other person, usually because they are geographically closer; (2) they report providing assistance often, and for this reason, should be included as partners in consultations on harm reduction strategies and overdoses prevention, and (3) they may be empowered by rendering assistance, which may have positive effects on network culture, norms, and structure.

### Limitations

In a true respondent-driven sample (RDS) with a number of recruitment generations, equilibrium can be realized when chain-referrals are continued for a minimum of six waves [20], allowing population means and percentages to stabilize. We had only a single wave to test the feasibility of asking participants detailed questions about a number of friends. Therefore, the study population cannot be assumed to represent the community of DTE and Abbotsford PWSD. Second, responses were self-reported and may be subject to the social desirability bias, though this was likely less than being interviewed by a health professional. Finally, because participants used aliases and nicknames for both themselves and their contacts or friends, we cannot be completely certain whether all contacts who have been named multiple times have been identified correctly. However, this was a systematic error across all participants and contacts, so the relative network sizes are likely valid.

Multiple studies have demonstrated considerable variation between the perception of social support and the actual supportive behavior [17, 26, 36]. Other studies have reported that the perception of social support has stronger influence on the health behavior of recipient than received support [12, 47]. We may have underestimated the effect of social support on harm reduction behavior, so that in fact it may be more important than reflected in the study. Nevertheless, this is one of the few studies on social support in PWUID and the only one in PWSD.

## Conclusion

We aimed to understand how network size and the social support provided by friends and contacts in these networks can promote harm reducing behavior among PWSD who may otherwise not be exposed to harm reduction initiatives. We demonstrated that the number of contacts in the social network of PWSD who were reported to provide tangible support increased the likelihood of the participant assisting friends or contacts and being trained in and/or carrying naloxone. We have shown the importance of social context surrounding PWSD, and how, given the resources and support, PWSD play a central role drugs in assisting others during overdoses.

## Data Availability

The data may be available under request to Dr. Ehsan Jozaghi.
